# Supplementary material for: Assessing the quality of antenatal corticosteroids in low- and middle-income countries: A systematic review
Source: PLoS One. 2020 Dec 3;15(12):e0243034. doi: 10.1371/journal.pone.0243034 (PMC7714108; doi:10.1371/journal.pone.0243034)
Supplement: S2 Appendix — (DOCX) [file pone.0243034.s002.docx]

## **S2 Appendix. Search Strategy**

| **MEDLINE**  **Date of Search:** 17 July 2019  **Number of Results:** 5386  **Search Strategy**   1. glucocorticoids/ 2. betamethasone/ or dexamethasone/ 3. (dexamethasone or betamethasone or corticosteroid*).mp. 4. (decaject or decameth or dexasone or dexpak or hexadecadrol or hexadrol or maxidex or methylfluorprednisolone or millicorten).mp. 5. (betadexamethasone or betamethasone or celeston or celestona or celestone or cellestoderm or flubenisolone).mp. 6. exp pregnancy/ 7. exp Obstetric Labor, Premature/ 8. (premature birth* or preterm birth* or antenatal care or antenatal or prenatal or prematur* or neonat* prematur*).mp. 9. (antenatal adj2 corticosteroid*).mp. 10. maternal health services/ or perinatal care/ or prenatal care/ 11. 1 or 2 or 3 or 4 or 5 12. 2 or 3 or 4 or 5 13. 6 or 7 or 8 or 9 or 10 14. 7 or 8 or 9 or 10 15. 11 and 13 16. 12 and 14 |
| --- |
| **EMBASE**  **Date of Search:** 17 July 2019  **Number of Results:** 4902  **Search Strategy:**   1. (adrenal cortex hormon* or adrenal cortical steroid or adreno cortical steroid adreno corticosteroid or adrenocortical hormon* or adrenocortical steroid or adrenocorticosteroid or cortic* steroid or corticosteroid agent or corticosteroid calcium or corticosteroid hormone or corticosteroid*).mp. [mp=title, abstract, heading word, drug trade name, original title, device manufacturer, drug manufacturer, device trade name, keyword, floating subheading word, candidate term word] 2. dexamethasone.mp. or dexamethasone sodium phosphate/ [mp=title, abstract, heading word, drug trade name, original title, device manufacturer, drug manufacturer, device trade name, keyword, floating subheading word, candidate term word] 3. corticosteroid.mp. 4. betamethasone acetate/ or betamethasone/ or betamethasone acetate plus betamethasone sodium phosphate/ 5. 1 or 2 or 3 or 4 6. exp perinatal care/ 7. ((matern* adj2 care) or (mater* adj2 health)).mp. [mp=title, abstract, heading word, drug trade name, original title, device manufacturer, drug manufacturer, device trade name, keyword, floating subheading word, candidate term word] 8. ((perinatal adj2 care) or (perinatal adj health)).mp. [mp=title, abstract, heading word, drug trade name, original title, device manufacturer, drug manufacturer, device trade name, keyword, floating subheading word, candidate term word] 9. exp prenatal care/ 10. ((antenatal adj2 care) or (ante natal adj2 care) or (prenatal adj 2 care) or (preterm adj2 birth)).mp. [mp=title, abstract, heading word, drug trade name, original title, device manufacturer, drug manufacturer, device trade name, keyword, floating subheading word, candidate term word] 11. 6 or 7 or 8 or 9 or 10 12. 5 and 11 |
| **CINAHL**  **Date of Search:** 27 July 2019  **Number of Results: 316**  **Search Strategy:**  1. Prenatal Care OR antenatal care OR Infant, Premature OR pre term birth  AND dexamethasone or betamethasone |
| **Global Index Medicus**  Date of Search: 25 July 2019  Number of Results: 4072  Search Strategy: (tw: (dexamethasone)) OR (tw: betamethasone) |
| **Medicines Quality Database (WHO)**  Date of Search: 25 July 2019  Number of Results: 244  Search Strategy: Geographic Locations; Africa, Asia, South America: Year; 2003 to 2017  Therapeutic Indication: “All”; Medicine Info; “All”; Dosage Form; “Injectable Ampoules”; Facility Information; “All’; Test Results Criteria; “Pass” “Fail” “Both”. |
| **International Pharmaceutical Abstracts**  Date of Search: 25 July 2019  Number of Results: 3581  Search strategy: noft(dexamethasone) OR noft(betamethasone) |
