# Supplementary material for: Assessing the quality of antenatal corticosteroids in low- and middle-income countries: A systematic review
Source: PLoS One. 2020 Dec 3;15(12):e0243034. doi: 10.1371/journal.pone.0243034 (PMC7714108; doi:10.1371/journal.pone.0243034)
Supplement: S3 Appendix — (DOCX) [file pone.0243034.s003.docx]

## **S3 Appendix. Quality Assessment of Studies using MEDQUARG tool^23^ ^36^**

| **MEDQUARG**  **Guidelines 12 Quality Criteria** | **UN Commission multi-country survey** | **India Ministry**  **of Health** |
| --- | --- | --- |
| 1) Timing and location of study clearly stated | 1 | 1 |
| 2) Definition of counterfeit / substandard med provided? | 1 | 1 |
| 3) Description of Type of outlet sampled | 1 | 1 |
| 4)Description of sampling design & sample size calculation | 0 | 1 |
| 5) Type and N of dosage units purchased from outlet | 1 | 0 |
| 6) Random sampling used? | 0 | 1 |
| 7)Info on who collected the sample (mystery shopper)? | 1 | 1 |
| 8) Packaging assessment performed? | 1 | 0 |
| 9) Statistical analysis described? | 0 | 1 |
| 10) Chemical analysis clearly described? | 1 | 1 |
| 11) Details on method validation | 1 | 0 |
| 12) Chemical analysis assessors blinded to packaging? | 0 | 0 |
| **Total Quality Score** | **8** | **8** |
