# Supplementary material for: Assessing the quality of antenatal corticosteroids in low- and middle-income countries: A systematic review
Source: PLoS One. 2020 Dec 3;15(12):e0243034. doi: 10.1371/journal.pone.0243034 (PMC7714108; doi:10.1371/journal.pone.0243034)
Supplement: S4 Appendix — (DOCX) [file pone.0243034.s004.docx]

## **S4 Appendix. Reported quality parameters for all available data**

| **Identification** | | **Manufacturing** | | | | **Sampling** | | | | |  | | **Testing** | | | | | | | |
| --- | --- | --- | --- | --- | --- | --- | --- | --- | --- | --- | --- | --- | --- | --- | --- | --- | --- | --- | --- | --- |
| **Study ID (1^st^ Author, publication year, type)** | **Country where conducted** | **Country**  **Were products were manufactured** | **Manufacturer**  **(Intl/Local)** | **Expiration Dates** | **Concentration** | **Time period when conducted** | **Sampling Method** | **Samples assessed for:** | **Total # of product samples obtained.** | **Type of Selling/**  **distribution point** | **Standard** | **Blinding of laboratory investigators to sample source** | | **Appearance** | **Identification** | **Prescense of Free Dexamethasone** | **pH** | **API%** | **% Failed Samples** |  |
| UN Commission on Life Saving Commodities 2015 Report | Kenya | China | Intl | Jan 14 to Jan 18 | 4mg/mL | Sep – Nov 2013 | Convenience sampling | Appearance, Identification, pH, API | 19 | Wholesaler Private | British Pharmacopeia | Not reported | | pass | pass | pass | 7.9 | 64.4 | 32.2% |  |
|  | Madagascar | China | Intl | Jan 14 to Jan 18 | 4mg/mL |  |  |  |  | Wholesaler Private |  |  | | Pass | pass | Pass | 8 | 99.6 |  |  |
|  | Madagascar | China | Intl | Jan 14 to Jan 18 | 4mg/mL |  |  |  |  | Wholesaler Private |  |  | | Pass | pass | pass | 8 | 84.6 |  |  |
|  | Nepal | India | Intl | Jan 14 to Jan 18 | 4mg/mL |  |  |  |  | Distributor Private |  |  | | Pass | pass | pass | 7.9 | 102.9 |  |  |
|  | Nepal | India | Intl | Jan 14 to Jan 18 | 4mg/mL |  |  |  |  | Public Treatment Centre |  |  | | Pass | pass | pass | 7.5 | 101.9 |  |  |
|  | Nepal | India | Intl | Jan 14 to Jan 18 | 4mg/mL |  |  |  |  | Public Hospital |  |  | | Pass | pass | 1.2% | 7.8 | 82.1 |  |  |
|  | Nigeria | China | Intl | Jan 14 to Jan 18 | 4mg/mL |  |  |  |  | Importer Distributor Private |  |  | | Pass | pass | pass | 7.2 | 88.8 |  |  |
|  | Nigeria | China | Intl | Jan 14 to Jan 18 | 4mg/mL |  |  |  |  | Importer Distributor Private |  |  | | Pass | pass | pass | 7.8 | 97.4 |  |  |
|  | Tajikistan | China | Intl | Jan 14 to Jan 18 | 4mg/mL |  |  |  |  | Importer Distributor Private |  |  | | Pass | pass | pass | 8.1 | 102.6 |  |  |
|  | Tajikistan | Russia | Intl | Jan 14 to Jan 18 | 4mg/mL |  |  |  |  | Importer Distributor Private |  |  | | Pass | pass | 0.7% | 7 | 92.8 |  |  |
|  | Tajikistan | China | Intl | Jan 14 to Jan 18 | 4mg/mL |  |  |  |  | Importer Distributor Private |  |  | | Pass | pass | pass | 7.9 | 100.6 |  |  |
|  | Tanzania | China | Intl | Jan 14 to Jan 18 | 4mg/mL |  |  |  |  | Importer Distributor Private |  |  | | Pass | pass | pass | 8 | 101.4 |  |  |
|  | Tanzania | China | Intl | Jan 14 to Jan 18 | 4mg/mL |  |  |  |  | Importer Central Medical Store Public |  |  | | Pass | pass | pass | 8.1 | 102.1 |  |  |
|  | Uganda | India | Intl | Jan 14 to Jan 18 | 4mg/mL |  |  |  |  | NGO |  |  | | Pass | pass | pass | 7.70 | 104.9 |  |  |
|  | Uganda | India | Intl | Jan 14 to Jan 18 | 4mg/mL |  |  |  |  | Wholesaler Private |  |  | | Pass | pass | pass | 7 | 101.9 |  |  |
|  | Vietnam | Vietnam | Local | Jan 14 to Jan 18 | 4mg/mL |  |  |  |  | Importer Distributor Public |  |  | | Pass | pass | pass | 7.8 | 105.1 |  |  |
|  | Vietnam | Vietnam | Local | Jan 14 to Jan 18 | 4mg/mL |  |  |  |  | Manufacturer Distributor |  |  | | Pass | pass | pass | 7.4 | 104.3 |  |  |
|  | Vietnam | Vietnam | Local | Jan 14 to Jan 18 | 4mg/mL |  |  |  |  | Manufacturer Distributor |  |  | | Pass | pass | 0.8% | 8.1 | 104.8 |  |  |
|  | Zimbabwe | India | Intl | Jan 14 to Jan 18 | 4mg/mL |  |  |  |  | Importer private |  |  | | Pass | pass | pass | 7.6 | 95.8 |  |  |
|  | Burkina Faso | N/A | N/A | N/A |  | N/A |  |  |  | N/A |  | N/A | | N/A | N/A | N/A | N/A | N/A | N/A |  |
| Ministry of Health and Welfare, Government of India | India | N/A | Intl/Local | Not reported | 4mg/mL | Feb 2013 | Random Sampling | Appearance, Identification, pH, API | <100 | Private and Public | India  Pharmacopeia. | Not Reported | | Pass | pass | N/A | pass | Not reported | Private (3.2%)  Public (20.2%) |  |
