# Supplementary material for: Assessing the quality of antenatal corticosteroids in low- and middle-income countries: A systematic review
Source: PLoS One. 2020 Dec 3;15(12):e0243034. doi: 10.1371/journal.pone.0243034 (PMC7714108; doi:10.1371/journal.pone.0243034)
Supplement: Attachment — Submitted filename: PONE-D-20-24549 feedback.docx [file pone.0243034.s005.docx]

PONE-D-20-24549
Assessing the quality of antenatal corticosteroids in low- and middle-income countries: a systematic review.
PLOS ONE

General comments:

The authors have conducted research on an original and interesting topic. The methodology of the systematic review is acceptable. Suggestions to consider for improving the quality and reporting of the study are outlined, below.

In the discussion, the authors have demonstrated knowledge of the medicine quality, in general. However, there seem to be few paragraphs of the discussion linked directly to the objective of the systematic review – assessing the quality of antenatal corticosteroids in low- and middle-income countries.

Detailed comments

| **Introduction** |  |
| --- | --- |
| In 2015, the World Health Organization (WHO) … for women *wo* are at risk of imminent preterm birth at 24 to 34 weeks’  gestation.(6) | Kindly correct typo ‘wo’ in the statement |
| We therefore aimed to identify and synthesise primary studies that examined the quality of antenatal corticosteroids (injectable dexamethasone or betamethasone) in LMICs. | Please could the authors clarify here whether the review focused on injectable corticosteroids (IM and IV or either) for all indications or for antenatal use, only  It will be useful to provide the indication of the intervention, i.e. management of preterm birth (as stated in ‘Study identification’) |
| **Methods** |  |
| General comments | For clarity, kindly re-arrange text in this section to follow the order recommended by the PRIMSA guidance, e.g. Study identification (eligibility criteria), Information sources, Search etc.  Kindly ensure referencing style is consistent, throughout |
| Methods for undertaking the systematic review refers to 2 citations ([20] and [32]) | Please clarify how [32] informed the methods  Please check the referencing style of [32] |
| Search terms, databases  and outcome measures were informed by a 2016 systematic review of oxytocin quality by Torloni et al. | For clarity, could the authors kindly state how the Torloni review informed their methodology? E.g., search terms; quality assessment; choice of analysis etc. |
| Study identification | It will be helpful to elucidate the study designs of interest.  Results indicate data from LICs and LMICs. Please clarify whether LICs are a subgroup of LMICs. If this is not the case, kindly address this as appropriate to avoid concerns with reviewer bias  Please check this sentence for comprehension, ‘*Specifically, any primary study that reported API, sterility or pH using valid laboratory methods.’* |
| Search strategy | The authors indicated that electronic searches were conducted in July 2019. Is it likely that recent relevant studies could have been missed? |
| Quality Assessment | The authors defined the criterion for good methodological quality. Please can you add the rating for remaining categories? |
| Data analysis | Kindly explain the term ‘failed samples’ as best as possible  Please could the authors indicate how data synthesis was undertaken? |
| **Results** |  |
| The authors of the UN Commission multi-country survey reported that dexamethasone was  manufactured in China (9 manufacturers), India (6), Russia (1) and Vietnam (3) | Please clarify what (6), (1) and (3) refer to , here |
| Primary and secondary outcomes | This is first mention of primary and secondary outcomes  (including appearance and extractable volume).  Please justify  To provide clarity, authors should kindly consider providing more detail for ‘failed’ or ‘low fail’ samples with respect to quality components examined (e.g. API, pH, sterility) and relevant assessment rating.  TABLES  Table 2 shows ‘*Appearance, Assay, pH,*  *Extractable Volume, Free Dexamethasone’* as Tests Performed. This is a bit confusing.  Table below Table 3 (page 19 of 23) has no title. It is unclear whether parts of the table are missing.  FIGURE  Figure 1: Kindly revise title to PRISMA flow diagram or Study selection  Numbers in the box – Full-text articles excluded with reasons up to 56 not 60. Please check |
| **Discussion** | Author present their findings in the light of previous relevant literature, perceptively and in detail but there is limited discussion of their findings (quality of studies, methodological differences etc.).  In the recommendations, authors missed an opportunity to highlight and link future research needs and the dearth of evidence relating to various quality components of injectable drugs. |
